# Supplementary material for: Are carbon emissions trading and green financial instruments synergistic? -Comprehensive quantitative research based on content analysis
Source: PLoS One. 2024 Mar 7;19(3):e0298601. doi: 10.1371/journal.pone.0298601 (PMC10919601; doi:10.1371/journal.pone.0298601)
Supplement: S1 Data — (ZIP) [file pone.0298601.s001.zip › Data/地级市Z_F工作报告.zip!/╔╜╢1⁄2/╫═▓⌐╩╨/└┤╫╘╣1⁄2╓┌║┼í╛┬φ┐╦╩2╛▌═°í┐_2010.docx]

各位代表：

　　现在，我代表市人民政府，向大会作政府工作报告，请予审议，并请各位政协委员和其他列席会议的同志提出意见。

　　一、2009年工作回顾

　　刚刚过去的一年，是新世纪经济发展最困难的一年，也是我们砥砺奋进，经受严峻考验，取得重大突破和显著成效的一年。在市委的坚强领导下，各级政府团结带领全市人民，以党的十七大和十七届三中、四中全会精神为指导，深入贯彻落实科学发展观和胡锦涛总书记、温家宝总理视察我省我市时的重要讲话精神，认真贯彻执行中央、省应对国际金融危机的一揽子计划和政策措施，着力保增长、调结构、促改革、惠民生，各项工作取得显著成绩，殷实和谐经济文化强市建设迈出坚实步伐。全年地区生产总值预计完成2450亿元以上，增长12%以上；地方财政收入完成128.77亿元，增长12.28%；城镇居民人均可支配收入和农民人均纯收入分别增长9.4%和9%。

　　一年来，我们坚定信心，攻坚克难，奋力实现了保持经济平稳较快发展的目标。2008年四季度以来，国际金融危机迅速蔓延，我市经济遭受严重冲击，外部环境急剧恶化，市场需求严重萎缩，经济增速快速回落，就业形势骤然严峻。面对历史罕见的冲击和挑战，市委、市政府坚决贯彻落实中央、省应对国际金融危机的决策部署，坚定不移地把保增长作为首要任务，组织动员全市上下采取过硬措施，全力遏制经济下滑态势。经过不懈努力，全市经济自去年二季度以来逐季回升向好，步入平稳较快发展的轨道。截至去年12月末，全市税收同比增长29.61%；规模以上工业销售收入、利税、利润分别增长16%、63%和65%；12月份全市工业用电量达到22.62亿千瓦时，接近危机前月用电量的最高水平。

　　一年来，我们抢抓机遇，化危为机，有效增强了即期增长与长远发展的动力和后劲。按照“出手快、出拳重、措施准、工作实”的要求，抓住国家实施积极的财政政策和适度宽松的货币政策的历史机遇，迅速策划、争取和组织实施了一大批项目，着力优化投资结构，增强投资拉动。规模以上固定资产投资增长25%。有232个项目列入中央、省预算内投资项目，107个项目列入省调控资金项目，290个项目列入省产业结构调整振兴规划，19个项目列入省扩内需急需建设项目，争取中央、省预算内投资及省调控资金24.91亿元。铁路规划建设取得重大突破，晋中南铁路沂源段等一批重大交通能源项目得到国家核准。建立和强化政府投资项目监察审计有效管理机制，使中央投资项目达到了“三个百分之百”的要求，全年政府投资项目工程造价审减额达4.92亿元，切实保证了政府投资的安全和效益。积极贯彻落实扩大内需的方针政策，全年实现社会消费品零售总额868亿元，增长19%。坚持把稳定外需放在更加突出的位置，千方百计保市场、保份额，外贸出口降幅逐步收窄，出口实物量保持稳定。全市利用外来投资190.1亿元，其中实际利用外资5.24亿美元，增长10%。“走出去”实现重大突破。检验检疫、海关、外汇管理等部门服务开放的功能进一步增强。

　　一年来，我们远近结合，标本兼治，推动产业结构调整和发展方式转变迈出了新步伐。在应对国际金融危机过程中，坚持把扩内需与调结构、转方式紧密结合起来，着力提高经济整体素质和效益。抓住机遇推动工业调整振兴。与国家、省规划相衔接，制定实施我市五大产业调整振兴规划和11个特色产业调整指导意见。我市有26家企业被省列入重点支持的工业企业名单，申报的功能玻璃特色产业基地、优质泵类产品生产基地、核电装备及配套材料工业园，分别获国家和省批准。鲁泰公司、金晶集团分别荣获省长质量奖及提名奖。新增中国驰名商标7件。产业布局调整力度加大，张钢和张店焦化煤气厂搬迁改造基本完成，中心城区东部化工区搬迁改造和南部建材区综合整治加快推进。着力提高自主创新能力。制定实施支持市高新区争创国家创新型科技园区系列政策措施，获批组建17家院士工作站，争取并组织实施一批国家、省重大科技专项。组织实施创新成长计划，全市重点扶持的30家创新成长型企业利税、利润分别增长71.61%、101.93%。高新技术产业产值占规模以上工业总产值的比重达到35.6%，比上年末提高2.1个百分点。连续七次荣获全国科技进步先进市称号。坚定不移地推进节能减排，电力工业通过“上大压小”，提前一年半超额完成“十一五”关停任务，水泥和碳酸钙行业实现脱胎换骨式的提升发展。预计全年可超额完成省政府下达的节能降耗任务指标，化学需氧量和二氧化硫削减量分别达到8964吨和24466吨。现代服务业发展步伐加快，兴业银行、招商银行及沃尔玛、佳世客、特易购等入驻淄博，淄博保税物流中心、齐鲁现代物流港等一批重大生产性服务业项目加快推进。全市服务业完成投资459亿元，比上年增长38%。预计全市服务业增加值占GDP比重达到33.7%。坚持不懈地夯实“三农”发展基础。全面落实中央强农惠农政策，全年用于“三农”支出达到34亿元，创历史记录。粮食生产连续七年实现增产。生态高效农业和农业产业化、标准化迈出新步伐。

　　一年来，我们统筹城乡，一体发展，促进生态和谐宜居城市建设取得了新突破。按照“全域淄博”理念，规划布局三大生态功能区，新城区控规覆盖率和乡镇总体规划编制率达到100%。坚持旧城区改造提升与新城区重点突破并举，高水平组织实施了一批重大城市基础设施和综合整治项目，总投资60亿元的28项城建重点工程完成年度计划，城市面貌焕然一新，城市功能大幅提升，人居环境明显改善。国道309张辛段改线等重点交通工程顺利竣工，市域路网结构进一步优化。抓住机遇策划实施了由政府主导、总投资200亿元以上的“两区三村”改造建设工程，完成年度投资45亿元，收到了扩内需、促统筹、拓空间、带产业的多重效果。加大环境保护工作力度，扎实推进新一轮碧水蓝天行动计划，城市污水集中处理率达到85%，全市13座污水处理厂的出水水质达到一级A排放标准。开工建设“引太入张”工程。发行15亿元治污减排公司债券，孝妇河、猪龙河、四宝山等重点治污减排和景观建设工程成效显著。全市各项绿化指标达到或超过全国绿化模范城市标准。秸秆禁烧和综合转化利用工作成果得到进一步巩固。小城镇建设完成投资60亿元，镇村面貌进一步改善。加快实施城乡同源同网饮水安全工程，新解决42.8万农村人口饮水安全问题。新建改造农村公路520公里。“百座小型病险水库”除险加固工程已有72座完成主体工程。

　　一年来，我们改革创新，积极作为，强化了科学发展的体制机制保障。大力拓展发展空间。稳步推进国土资源管理改革试点，在全省率先完成新一轮土地利用总体规划编制及上报工作，土地执法监察工作跃居全省前列，为依法争取发展空间赢得了主动权。通过集约利用、淘汰落后等多种方式盘活存量土地1.5万余亩，腾出环境容量350万吨标准煤。进一步深化投融资体制改革。加强政银企合作，与四大国有银行签署三年增加600亿元贷款协议，年末全市金融机构新增贷款余额321.67亿元，是2008年的2.17倍。加强政府投融资体系建设，为高效筹集城乡建设资金奠定了体制保障基础。淄博股权托管中心和齐鲁股权交易中心正式挂牌运营，新增境内外上市公司2家、股权交易市场挂牌交易企业11家，全市上市企业通过资本市场融资66.7亿元。安排资金专项用于支持担保体系建设，全市担保机构发展到65家，新增小额贷款公司2家。政府信息资源共享加快推进，城市一卡通系统建成投用。促进区域协调发展。支持三个老工业区和沂源、高青加快发展以及援川、援藏工作取得新进展。

　　一年来，我们统筹兼顾，保障民生，促进了改善民生与扩大内需的有机统一。努力克服金融危机导致的阶段性财政困难，确保各项民生政策兑现落实。政府为民办的18件实事顺利实施。积极落实稳定和扩大就业的各项政策，新增城镇就业再就业11.2万人，转移农村劳动力7.3万人，城镇登记失业率控制在2.83%。提高了企业养老、失业、医疗和工伤四项社会保险参保人员待遇以及新农合筹资、城乡低保和社会救助标准，扩大新农保试点范围，全部解决了国有(集体)破产关闭企业退休人员的医疗保险问题。国家保障性住房政策得到全部落实。加大教育投入，加快高校资源整合，开工建设市特教中心、张店八中等一批新建学校，中小学校舍安全、农村初中校舍改造、学校标准化建设“三大工程”完成年度任务，免除43.8万名义务教育阶段学生杂费2.3亿元，教育教学水平不断提高。千方百计筹措资金，周密组织部署，医药卫生体制改革稳步启动。完成4处市县级医院、19所乡镇中心卫生院、2个城市社区卫生服务中心和1268处村卫生室改造建设。手足口病、甲型H1N1流感等重大传染病防控有序有效。稳定低生育水平，人口自然增长率控制在3‰以内。加强公共文化服务体系建设，文化信息资源共享工程扎实推进，新建扩建基层文化站点340个。成功协办全运会和亚足联亚青赛等高层次体育赛事，竞技体育水平不断提高，全民健身活动蓬勃开展。以生态文明建设为突破口，广泛开展群众性精神文明创建活动，荣获全国创建文明城市工作先进市称号。着力做好青少年工作，被评为全国未成年人思想道德建设工作先进城市。深入实施固本强基维稳工程，完善社会矛盾预防调处机制，扎实做好信访工作，加强社会治安综合治理，切实抓好安全生产，荣获全国社会治安综合治理优秀市称号。应急管理体系建设进一步完善，预防和处置突发事件能力明显增强。食品药品安全和质量监管不断加强。国防动员、人民防空、民兵预备役建设、双拥共建和优抚安置工作成效显著，军政军民团结进一步巩固。妇女儿童权益得到有效保护。慈善、残疾人、仲裁、老龄及红十字事业持续发展，民族宗教、外事、侨务、对台、史志、档案、防震减灾、气象、统计、新闻出版、广播电视等工作取得新成绩。各级政府按照为民、务实、清廉的要求，扎实开展深入学习实践科学发展观活动，加快政府职能转变，加强政府法制建设，强化行政监察、审计和工作督查，全面落实廉政建设责任制，政府自身建设迈出新步伐。

　　各位代表！回顾过去一年的工作，全市上下在市委的坚强领导下，不仅努力实现了保增长、保民生、保稳定的目标，而且在一些事关全局和长远的重大问题和关键领域实现了新的突破。成绩的取得，得益于党中央、国务院和省委、省政府的科学决策和亲切关怀，胡锦涛总书记、温家宝总理亲临我市视察，对我市工作提出了殷切希望，给全市人民极大鼓舞和有力鞭策。成绩的取得，得益于市委的坚强领导，得益于市人大、市政协和社会各界的有效监督、大力支持，得益于全市人民的团结奋进、务实拼搏。在此，我代表市人民政府，向全市人民，向各民主党派、工商联、各人民团体和社会各界人士，向驻淄部队、武警官兵、政法干警和中央、省驻淄单位，向所有关心支持淄博发展的同志们、朋友们和海内外友好人士，表示衷心的感谢和崇高的敬意！

　　在充分肯定成绩的同时，我们也清醒地看到，我市经济社会发展还面临一些困难和问题。主要是：经济回升的基础还不牢固，财政收支矛盾突出，调结构、转方式任重道远，资源环境约束比较突出，结构性就业难的问题短期内难以完全解决，政府工作在职能转变、干部作风、办事效率等方面还存在一些问题。对这些矛盾和问题，我们要采取更加扎实、有效的措施，努力加以解决。

　　二、2010年政府工作的指导思想和任务目标

　　今年是实施“十一五”规划的最后一年，做好今年工作，对于夺取应对国际金融危机冲击全面胜利、保持经济社会平稳较快发展、为“十二五”规划启动实施奠定良好基础至关重要。总体上看，中国经济在全球率先实现回升向好，世界经济有望实现恢复性增长，今年我市面临的发展环境将好于去年。但国际金融危机的深层次影响依然存在，国内经济回升的内在动力仍然不足，仍存在较大的不确定性。对此，我们既要坚定发展的信心，又要充分估计面临的困难和挑战，进一步增强忧患意识和风险意识，更加周密地做好应对各种复杂局面的准备，努力在新起点上把改革发展稳定各项工作做得更好。

　　今年政府工作的指导思想是：以邓小平理论和“三个代表”重要思想为指导，认真贯彻落实党的十七大和十七届三中、四中全会精神，深入贯彻落实科学发展观和胡锦涛总书记、温家宝总理视察我省我市时的重要讲话精神，坚定不移地走内涵发展的路子，着力保持经济社会平稳较快发展，着力加快经济结构调整和发展方式转变，着力推进城乡统筹发展，着力深化改革扩大开放，着力保障和改善民生，不断开创殷实和谐经济文化强市建设新局面。全市经济社会发展的主要建议目标是：地区生产总值增长12%，地方财政收入增长12%，规模以上固定资产投资增长20%，社会消费品零售总额增长16%，外贸进出口与实际利用外资分别增长5%和10%，城镇居民人均可支配收入和农民人均纯收入均增长8%左右。万元GDP能耗下降6%，全面完成省政府下达的“十一五”期间二氧化硫、化学需氧量减排任务，完成省政府下达的二氧化碳年度减排任务，人口自然增长率控制在5.5‰以内，城镇登记失业率控制在4%以内。上述指标，既考虑与“十一五”规划目标相衔接，又综合考虑发展的宏观环境、支撑条件以及发展趋势等因素。在生产总值、财政收入等指标安排上，留有一定余地，主要是为了引导各方面把工作重点放到转方式、调结构、提高发展的质量和效益上来。

　　三、保持经济平稳较快发展

　　当前我市经济发展正处于关键时期，回升向好的局面需要倍加珍惜和巩固，必须一以贯之地把保持经济平稳较快发展作为关系全局的重要任务，始终做到思想上不懈怠，目标上不动摇，工作上不松劲，为调结构、转方式、惠民生、保稳定夯实基础。

　　(一)努力保持投资合理增长。充分发挥投资对拉动经济增长、促进结构调整的重要支撑作用，进一步优化投资结构，引导资金重点投向经济社会发展的关键领域和薄弱环节。集中抓好总投资1800亿元的565个重大项目建设，确保完成年度投资600亿元以上。深入开展项目集中推进活动，健全投资者所期盼、项目建设所必需的项目建设推进机制，加强立项审批、土地供应、资金保障的协调推进和基础建设条件、公共设施配套的跟踪保障。深刻把握国家、省投资支持重点和政策调整变化趋势，在战略性新兴产业、自主创新、环境保护以及民生领域继续策划和储备一批项目，加大对上争取力度，确保项目建设接续有力。进一步优化投资环境，拓宽投资领域，激活民间投资。

　　(二)着力扩大消费。充分发挥消费对经济增长的拉动作用，引导和改善居民消费预期，进一步优化消费环境，提高居民消费能力和意愿。一是着力增强居民消费能力。千方百计增加城乡居民收入，进一步提高居民收入在国民收入中的比重和劳动报酬在初次分配中的比重。认真落实最低工资制度，落实各项保障优抚政策，提高群众特别是低收入群众的消费能力。二是积极培育消费增长点。全面落实国家鼓励消费的各项政策，促进大宗商品消费和消费结构升级。积极发展社区商业、物业、家政等便民消费，扩大旅游休闲、文化娱乐、体育健身等服务消费，加强对“两区三村”改造后群众消费需求的引导，加快培育形成新的消费增长点。三是完善城乡流通市场体系。深入实施“万村千乡”市场工程，加强标准化农家店、连锁经营物流配送中心、社区连锁店和农产品批发市场建设，降低流通成本，方便群众消费。四是切实改善消费环境。加强市场价格和关系群众生命健康安全的重点产品的监管，严厉打击扰乱市场秩序、假冒伪劣、价格欺诈、虚假广告等违法犯罪行为，维护消费者合法权益，营造安全放心的消费环境。

　　(三)提高开放型经济水平。在努力扩大内需的同时，绝不放松挖掘外需增长潜力。一是加快外贸发展方式转变。深入实施市场多元化战略，巩固传统市场，加大新兴市场开拓力度。发挥政策集成效应，集中扶持优势企业、出口大户、重点产品扩大出口。调整优化出口产品结构，努力扩大高附加值、高技术含量产品及自主品牌产品出口。抓好市高新区省级服务外包基地建设，提高服务贸易比重。引导企业积极应对国际贸易摩擦，有效规避出口风险。支持进口先进技术装备、关键零部件和紧缺物资，增加战略资源进口和储备。二是提高招商引资的质量和水平。创新招商思路，大力实施产业招商、产权招商和大企业招商，扩大与国内外大企业、大财团的战略合作，争取在股权融资、境外上市融资领域实现更大突破。正确引导外商投资方向，鼓励外来投资投向高端高质高效产业领域。积极争取世行、亚行优惠贷款，做好国外贷款项目实施推进和筛选储备工作。三是积极实施“走出去”战略。支持企业到境外并购优质资产、品牌、技术和营销网络，深入开展能源原材料合作开发。抓好在建的境外产业转移项目。继续引导相关行业企业到资源环境更为适宜的地区建立生产基地，寻求新的发展空间。继续抓好对外工程承包和劳务合作，大力发展高端劳务。

　　(四)提高财政经济质量和效益。强化效益优先观念，努力依法聚财、科学理财、有效用财。加强税收征管，加大综合治税力度，堵塞税收流失漏洞。进一步加强非税收入管理，抓好国有资产和政府资源监管运营。优化财政支出结构，创新财政支持发展、培植财源的方式，加大对优质财源产业项目以及对信用担保体系、创新成长型企业、战略性新兴产业等重点领域和关键环节的支持力度。严格控制一般性支出，集中财力保重点、保民生。严肃财经纪律，勤俭办一切事业。

　　四、推进发展方式转变和结构调整

　　无论是克服当前困难，还是谋划长期发展，都对转方式、调结构提出了新的更为迫切的要求，必须坚持把结构调整作为经济社会发展的主线，把节能降耗作为经济社会发展的关键，把环境保护作为经济社会发展的命门，下决心打好转方式、调结构这场硬仗。

　　(一)推动工业结构优化升级。加快培育壮大高端高质高效产业，努力抢占发展制高点，扩大市场占有率，形成核心竞争力。一是大力培植战略性新兴产业。制定实施我市战略性新兴产业发展行动方案，完善政策支持，加大投入力度，发挥比较优势，大力发展新材料、精细化工、信息、医药、汽车及机电装备等产业，力争经过三至五年的努力，使新兴产业在工业经济中的比重由目前的30%左右提高到50%以上。积极争取国家和省新兴产业、高端产业发展专项基金，健全有利于新兴产业快速成长的投融资机制，发挥省级以上开发区的平台作用，促进战略性新兴产业尽快形成规模集聚效应。二是加快传统产业升级改造。扎实组织实施重点产业调整振兴规划，重点抓好290个省调整振兴规划项目和390个市调整振兴规划项目。大力推进技术改造，加快“5年1500亿元”技改计划的实施，保持技改投入稳定增长的态势，年内力争完成技改投资500亿元以上，大幅度提高传统产业的技术标准和工艺装备水平。加速推进工业化和信息化融合，培植20家信息化示范企业，推广50个“两化融合”重点项目，提高自动化、智能化和管理现代化水平。三是促进重点企业和中小企业协调发展。建立科学的企业绩效评估体系，引导企业积极调结构、转方式。继续开展夺星创优争百强活动，集中优质资源，支持列入省350户重点工业企业名单的26家企业加快发展，支持50家创新成长型企业跨越发展，充分发挥重点企业在应对危机挑战、产业调整振兴、重大技术创新等方面的龙头带动作用。认真贯彻国家、省促进中小企业发展的政策措施，切实帮助中小企业解决融资难、担保难问题。

　　(二)大力发展现代服务业。深入研究服务业业态运行规律，针对不同业态高质高效运行的特点和外部条件需求，分门别类优化运行环境，特别对既服务生产，又对经济结构调整、节能减排、财政增收具有重大支撑作用的企业和项目，进一步加大支持力度，破除发展瓶颈，促其健康快速发展，努力提高服务业对地方经济发展的贡献率。加快淄博保税物流中心、齐鲁现代物流港等大型高端物流项目的建设进度，尽快将建设现代“旱码头”的规划变为现实。加强现代商贸流通设施的规划布局与建设，培育壮大一批有竞争力的专业市场和大型零售企业。大力发展文化旅游业，发挥优势，整合资源，策划精品项目，打造“齐风陶韵、生态淄博”旅游产业品牌。抢抓机遇，做大做强金融证券、保险服务等产业，提高城市金融服务功能和产业自身质量效益。规范和策划并重，促进房地产业、物业管理、家政服务等产业健康发展。高度重视发展会展经济，创新办好陶博会。

　　(三)强化农业基础地位。坚持城乡统筹发展，认真落实各项强农惠农政策，把大力发展现代农业作为转变经济发展方式的重要任务，毫不放松地抓好农业农村工作。一是推动传统农业向精准、优质、高效农业转变。全面落实各项农业补贴政策和最严格的耕地保护政策，搞好农业综合开发，提高农业综合生产能力。在稳定粮食生产的基础上，抓好牧、菜、果等主导产业的标准化、基地化建设，培植优质高效农业品牌。大力发展都市农业，布局建设以中心城区为重点的都市农业聚集区，切实提高农业的经济效益、社会效益、生态效益和景观效益。加快生态有机农业发展，支持沂源县、博山区率先整建制建设成为有机农产品生产区，建设淄河流域11个乡镇整建制生态有机农业示范区。搞好基层农技推广体系改革与建设示范县试点。二是着力加强农业农村基础设施和环境建设。继续加大对农业农村的投入。全面完成“百座小型水库”除险加固任务。加快城乡供水同源同网工程和农村饮水安全工程建设，完善村村通油路工程和乡村道路管理养护机制。巩固秸秆禁烧和综合利用成果，继续实施户用沼气进万家工程，推进循环农业示范市建设上水平。三是推动农业农村体制机制创新。稳定和完善农村基本经营制度，在依法自愿有偿流转的基础上发展多种形式的适度规模经营。基本完成集体林权制度改革任务。大力发展农民专业合作社。加快农村金融改革步伐。继续深化农村综合改革。

　　(四)提高自主创新能力。启动实施技术创新工程，着力抓好重点领域重大产业技术创新项目，力争200个项目列入省技术创新项目计划，完成1000项重点行业领域的新产品、新技术研发。推进与百所高校、科研院所的战略合作，加大院士工作站、博士后工作站、重点实验室、省级以上企业研发机构等高层次产学研合作载体建设力度。继续承办好新材料技术论坛。支持市高新区加快争创国家创新型科技园区，推动区域创新资源整合，提高公共创新平台和公共服务平台建设运营水平，努力把高新区建设成为引领发展方式转变的现代科技城。加强知识产权保护，继续深入实施品牌战略和技术标准战略。加强高技能人才队伍建设，实施“高层次人才智力引进及培养工程”，加快海外高层次人才创新创业基地建设。

　　(五)切实抓好节能减排。强化措施、强化责任、强化考核，确保全面完成“十一五”节能减排任务。严格执行区域能源消费总量、污染排放总量控制制度和新增能耗等量替代制度，对区域能源消费总量实行预警控制告知。严格控制新上“两高一资”项目，加快淘汰落后产能，全面完成立窑水泥产能淘汰任务，完成钢铁行业落后产能年度淘汰计划，加快推进淘汰高耗能变压器、锅炉和电机系统改造等三大节能改造工程，确保完成淘汰改造任务。积极推行能源合同管理。深入实施建筑节能等“十大节能工程”和第三批“三个节能30项”工程，大力推广应用节能新技术、新工艺和新设备。着力调整优化产业布局，促进产业向园区集中，加快中心城区东部化工区、南部建材区和四宝山地区等重点区域产业布局调整步伐。抓住中石油泰青威天然气管线横贯我市的有利条件，优化能源结构，发展低碳经济，提升传统产业发展水平。大力发展循环经济，按照国家和省统一部署，启动二氧化碳减排工作。提高节能减排标准，对各类环境违法行为，继续实行顶格处罚，不断巩固扩大生态文明建设成果。

　　(六)加快推进新型城镇化。抓住新型城镇化快速推进的重大历史机遇，布局实施总投资270亿元的280项城镇化重点项目，全面落实“四个原则”、“八个意识”，加快新型城镇化进程，提升新型城镇化水平。一是做大做强做美做畅中心城区。坚持新城区开发与老城区改造并重，内涵提升与外延扩展相结合，加快建设整体功能突出、生态环境优良、现代气息浓郁的“双百中心城区”，提升中心城区辐射带动功能。进一步加大老城区改造力度，切实加快推进新城区建设，全面高质量完成全市城镇化工作会议确定的中心城区72项城建重点工程年度建设任务。二是加快构筑市域一体化发展的城镇组团体系。在全市总体规划框架指导下，正确处理“四个层级”的承接互动关系，搞好各区县与中心城区的衔接，重大基础设施统一规划、共建共享，逐步把次中心城区建设成富有特色、功能完善的中等城市规模。积极发展壮大中心镇、中心村。选择多个区位优势明显、产业基础好、城镇化水平高的经济强乡镇或工业集中区作为连接点，提升其承接和带动功能。继续下大气力抓好“两区三村”建设，力争年底前完成大部分改造工程，配套提升道路、管网、社区服务等设施，同步整治、改造周边环境，确保达到拉动经济增长、拓宽发展空间、推动社会建设、促进民生改善等多重效果。稳步推进户籍制度改革，吸纳农村人口向城镇转移。三是进一步提升城镇综合承载能力。进一步完善市域“六纵九横”和区际“三纵三横”干线公路网，重点抓好中心城区“大外环”、薛馆路沂源段、庆淄路高青段等干线公路的改造建设，提升完善“大十字”公交体系，形成干支相连、区域协调、城乡通达、顺畅便捷的现代综合道路交通网络。集中力量搞好晋中南铁路沂源段等重大铁路项目建设。抓住发展特高压电网和“外电入鲁”的机遇，推进重点电网工程建设，优化电网结构。继续抓好“引太入张”和“清水润城”工程，开工建设南水北调淄博段工程，进一步加快城区雨污管网改造，完善城乡垃圾一体化处理体系，组织实施博山——沂源输气管线建设。完善城市防灾减灾基础设施。四是大力推进生态建设。突出园林城市特色，重点实施以太河水库、萌山水库、孝妇河、淄河和四宝山为重点的“两库两河一山”生态建设工程。扎实开展城乡环境绿化，重点抓好城区精品园林建设和城区间绿色通道衔接，继续组织实施荒山绿化、“两沿三环”绿化和农田林网建设，形成大生态、大绿化格局。全面启动国家环保模范城市创建工作，深入实施大气污染治理再提高工程，着力改善水环境质量，全面完成第二轮碧水蓝天行动计划，力争三年内实现环保模范城创建目标。五是切实提高城市管理运营水平。加强城乡环境综合整治，突出抓好济青高速沿线、胶济铁路沿线、昌国路沿线和火车站广场及其周边地区治理，抓好城乡结合部、交通干道沿线、乡镇驻地、农村等重点部位的综合整治，改善城乡环境，提升城市形象。积极创新融资、建设、管理、运营机制，有效整合各类资源，形成城市建设投融资良性循环。加快建立城市管理长效机制，推进数字化城市建设，实现城市管理的精细化、规范化、科学化。

　　(七)深化经济体制改革。扎实推进重点领域和关键环节改革，努力为调结构、转方式提供有力的体制机制保障。一是进一步深化企业改革。规范企业法人治理结构，鼓励企业制度创新、管理创新。积极支持企业上市融资及再融资，争取有3家以上企业在境内外上市。继续加强淄博齐鲁股权交易中心与天津股权交易所的战略合作，力争有更多符合条件的企业实现挂牌交易。充分发挥淄博股权托管中心的作用，规范非上市股份公司运作。二是推进投融资体制改革。抓住国家继续实施适度宽松的货币政策的有利时机，加大政银企合作力度，进一步优化金融生态环境，为金融机构优化信贷结构、增加信贷投放创造条件。继续支持外埠银行在我市设立分支机构。抓好中小企业贷款专营机构建设，规范发展村镇银行、小额贷款公司，支持融资租赁、创业风险投资加快发展。加强信用担保体系建设，帮助企业完善贷款基础条件，切实提高中小企业融资能力。三是推进资源性产品价格改革。积极稳妥地推进资源性产品价格改革。继续深化电价改革，争取开展用电大户与发电企业直接交易试点和替代发电试点，落实差别电价政策。开展环境容量有偿使用和排污权交易试点工作。继续规范和完善各种矿产资源的价格调节基金征收制度。四是大力拓宽发展空间。积极扎实地组织做好国土资源管理改革试点工作，用足用好城乡建设用地增减挂钩政策，结合推进新型城镇化、“两区三村”工程、淘汰落后以及相关片区、企业的搬迁改造等，强化行政责任，加大政策激励约束，努力在拓展有形土地空间和无形环境容量两个方面取得大的进展。五是积极对接全国、全省重大发展战略布局。抓住黄河三角洲开发上升为国家战略的机遇，加强与国家、省重大区域发展战略的对接，努力为经济发展争取更大空间。进一步落实促进老工业区和市高新区科学发展的政策措施，加大支持高青、沂源发展力度，壮大区县经济实力。进一步做好援川、援藏工作。

　　五、着力保障和改善民生

　　把惠民生作为转方式、调结构、扩内需的出发点和着力点，坚持以人为本、民生优先，大力加强社会建设，不断扩大公共服务，切实解决人民群众最关心最直接最现实的利益问题，维护社会和谐稳定。

　　(一)做好就业和社会保障工作。一是坚持实施积极的就业政策。加快建设创业型城市，力争全市以创业带动就业的人数占城镇新增就业人数的比重有较大幅度提高。建立公共投资带动就业增长的综合评价机制，挖掘政府投资和大项目投资带动就业增长的潜力，重点解决大中专毕业生、城市困难群体和农民工就业问题。加强劳动用工管理，规范经济性裁员，稳定就业岗位。全年实现就业再就业7.6万人，转移农村劳动力6.2万人。二是不断提高社会保障水平。全面落实各项社会保险提标政策，确保及时足额发放。统筹城乡社会保障制度建设，积极开展城镇居民养老保险试点，扩大新型农村基本养老保险试点范围。三是完善城乡社会救助制度。进一步提高城乡低保和农村五保供养标准。实施残疾人托养安养工程和贫困残疾儿童康复救助计划。加强保障性住房建设，全面完成省下达的保障性住房建设任务，不断完善廉租住房货币化补贴政策。

　　(二)深化医药卫生体制改革。医药卫生体制改革是一项惠及城乡全体居民的重大改革，要深入调研，周密组织，稳步推进。一是加快推进医药卫生体制五项改革。出台配套政策，有序加快改革进程。力争全市60%的城市社区卫生服务机构和区县政府举办的基层医疗卫生机构实施基本药物制度。启动公立医院改革试点工作。大力支持中医药事业发展。二是提高城乡居民医疗保障水平。建立城镇医疗保险门诊统筹制度，全面落实已出台的提高大额医疗救助基金最高支付限额和城镇居民医保一级医院报销比例、门诊慢性病限额优惠等政策。提高新型农村合作医疗筹资标准、财政补助标准和最高支付限额。在将城乡低保和农村五保户纳入医疗救助范围的基础上，逐步将因病致贫等特殊困难家庭纳入医疗救助范围，逐步降低或取消医疗救助起付线，合理设置封顶线。三是加强疾病预防控制和卫生机构建设。加快推进市中心医院病房大楼等重点项目建设，完成县级医院和城乡社区医疗服务机构规划建设年度任务，加强乡镇卫生院、城市社区卫生服务中心医疗力量，提高医疗服务水平。继续实施重大和基本公共卫生服务项目，做好重大传染病防治工作。

　　(三)繁荣发展教育、文化等社会事业。坚持教育优先发展，深入实施素质教育，全面提高教育教学质量和办学水平。完善义务教育经费投入保障机制，全面落实家庭经济困难学生资助政策。加快新建学校建设进度，完成中小学校舍安全、农村初中校舍改造和学校标准化建设“三大工程”年度计划，抓好农村中小学“两热一暖一改”工程推广。优化区域职教资源配置，大力发展职业教育。加强社会主义核心价值体系建设，广泛开展群众性精神文明创建活动，努力争创全国文明城市。抓好网络视听领域的规范管理，提升乡村少年宫建设水平。繁荣发展文化事业和文化产业，实施文化惠民工程，加快新区文化中心和乡镇综合文化站建设，逐步完善公共文化服务体系。繁荣发展新闻出版、广播影视、文学艺术事业，加强文化遗产保护与利用。深化文化体制改革，深度开发以齐文化为代表的优秀传统文化资源，加快市域省级及国家历史文化名城的申办步伐，扶持发展会展、文博、创意、动漫等文化产业，提升淄博文化的影响力。继续强化人口和计划生育工作，稳定低生育水平。深入开展爱国卫生运动。积极支持青少年、红十字、老龄、慈善事业发展。加强妇女儿童权益保护。加强国防教育和民兵预备役建设，落实各项优抚政策，争创全国双拥模范城“七连冠”。进一步做好统计、民族宗教、外事、侨务、对台、仲裁、史志、档案、气象、人防、防震减灾等工作，促进各项事业协调发展。

　　(四)切实维护社会和谐稳定。坚持把维护社会稳定作为第一责任，深入实施固本强基维稳工程，重点抓好社会矛盾化解、社会管理创新和公正廉洁执法，扎实推进“平安淄博”建设。加强社会治安综合治理，严厉打击各类违法犯罪活动。严格落实政府监管和企业主体“两个责任”，毫不放松地抓好安全生产。加强民主法制建设，争创全国法制宣传教育先进城市。加强食品药品安全监管，保障人民群众生命健康。加强应急管理平台体系建设，提高预防和处置突发事件的能力。全面实施社区建设三年规划，搞好社区“五项工程”建设，夯实社会管理基础。

　　(五)全力做好第二十二届省运会承办工作。突出“相约生态淄博、共创和谐省运”主题，坚持举市办省运、创新办省运、开放办省运、节俭办省运，重点抓好场馆建设、开闭幕式大型文体活动、赛会运行、环境整治、服务保障、安全保障、舆论宣传等关键环节，以一流的场馆设施、一流的组织服务、一流的竞赛成绩、一流的城市形象，充分展示淄博经济社会发展的辉煌成就和全市人民的良好精神风貌，努力把省运会办成一届高水平、有特色的体育盛会，不辜负省委、省政府的重托和全市人民的期望。以承办省运会为契机，深入开展全民健身运动，不断提高竞技体育水平，大力发展体育产业，努力跻身全国体育综合实力先进行列，推动体育强市建设实现新突破。

　　六、切实加强政府自身建设

　　主动顺应形势发展的新变化和人民群众的新期待，深入推进服务型政府建设，切实提高推动科学发展、和谐发展的能力和水平。一是加快转变政府职能。完成市、区县政府机构改革任务，进一步深化行政管理体制改革，减少和规范行政审批事项。加强电子政务建设，降低行政成本，提高行政效率。切实加强社会管理和公共服务职能，注重从制度上消除发展瓶颈，破解发展难题，优化发展环境，提供发展动力。二是提高依法行政水平。加强行政执法能力建设，规范行政执法自由裁量权，强化行政执法责任制和责任追究制，切实保护公民、法人的合法财产和正当权益。推进政府信息资源公开与共享，保障人民群众的知情权、参与权、表达权和监督权。促进行政问责的制度化、规范化，加大对行政不作为和乱作为的问责力度。自觉接受人民代表大会及其常委会的法律监督和工作监督，积极支持人民政协履行政治协商、民主监督、参政议政职能，认真办理人大代表建议和政协委员提案。广泛听取各民主党派、工商联、各人民团体和人民群众的意见和建议，高度重视社会公众监督和新闻舆论监督。三是大力加强作风建设。政府全体工作人员特别是各级领导干部，要牢固树立科学的发展观和正确的政绩观，多干打基础、利长远的事，多为群众办实事、解难题，努力创造经得起实践和历史检验的政绩，坚决杜绝劳民伤财的形式主义。大力弘扬认真专业务实政风，深入基层一线，问政于民、问需于民、问计于民，使政府决策更加符合人民意愿。健全完善目标管理责任制，把经济社会发展目标逐级分解，时间倒推、工期倒排、任务倒逼，一级抓一级，层层抓落实。始终保持艰苦奋斗的优良作风，严格控制公务支出，坚决制止铺张浪费，建设节约型政府。四是提高拒腐防变能力。深入开展廉政建设和反腐败斗争，强化监察、审计等专门监督，加强建筑工程、土地交易、产权交易、政府采购、医药购销、政府投资项目监管等重点领域的源头控制，严肃查处各类违法违规案件。各级政府和各部门负责人要严格落实廉政建设责任制和廉洁自律规定，勤勉尽责，秉公用权，切实维护政府的良好形象。

　　编制好“十二五”规划是今年政府工作的一项重要任务，我们将高度重视，周密部署，精心组织，深入调查研究，广泛征求意见，充分集中民智，努力提高规划编制的前瞻性、科学性和指导性。

　　各位代表！做好今年工作，任务艰巨而繁重，责任重大而光荣。让我们紧密团结在以胡锦涛同志为总书记的党中央周围，在中共淄博市委的坚强领导下，解放思想，开拓创新，充满激情，科学创业，全力推动经济社会又好又快发展，在建设殷实和谐经济文化强市的征程上迈出更加坚实的步伐！
